# Streptococci as the new dominant aetiological factors of mastitis in dairy cows in north-eastern Poland: analysis of the results obtained in 2013–2019

**DOI:** 10.1186/s13620-020-00181-z

**Published:** 2021-01-04

**Authors:** E. Kaczorek-Łukowska, J. Małaczewska, R. Wójcik, K. Naumowicz, A. Blank, A. K. Siwicki

**Affiliations:** 1 Department of Microbiology and Clinical Immunology, Faculty of Veterinary Medicine, University of Warmia and Mazury in Olsztyn, Oczapowskiego 13, 10-719 Olsztyn, Poland; 2 Department of Pathophysiology, Forensic Veterinary Medicine and Administration, Faculty of Veterinary Medicine, University of Warmia and Mazury in Olsztyn, Oczapowskiego 13, 10-719 Olsztyn, Poland

**Keywords:** Cattle, Mastitis, *Streptococcus*, *Staphylococcus*

## Abstract

The objective of our study was to evaluate prevalence of selected bacterial and fungal pathogens of mastitis in dairy cattle in north-eastern Poland. Our study was conducted from 2013 to 2019 in 1,665 clinically and sub-clinically infected quarter milk samples (2013, *n* = 368; 2014, *n* = 350; 2015, *n* = 290; 2016, *n *= 170; 2017, *n* = 173; 2018, *n* = 224; and 2019, *n* = 90). The isolation and identification of the pathogens were performed in keeping with generally accepted microbiological procedures. In 2013, mastitis was most commonly caused by *Staphylococcus aureus* (24%), *Streptococcus spp.* (22%), *Streptococcus agalactiae* (12%) and coagulase-negative staphylococci (11%). In 2014, the most common pathogens were *Streptococcus spp.* (25%), *Staphylococcus aureus* (18%) and coagulase-negative staphylococci (10%); in 2015, 2016, 2017, 2018 and 2019, *Streptococcus spp.* (from 39–49%) were the most frequent strains isolated from the quarter milk samples. Other pathogens were isolated occasionally (below 15% in all years). In conclusion, the role of environmental bacteria has been gradually increasing in the Warmia Province. The importance of infectious pathogens has been decreasing, indicating the efficacy of the applied preventive programmes and a need for the development of new programmes targeting environmental pathogens.

## Introduction

Mastitis is one of the causes of substantial economic losses in dairy cattle production in Poland and many other countries. These losses are associated with reduced milk production performance, costs of veterinary care or the necessity of elimination of chronically ill animals from the herd [1–3]. Considering the occurrence/severity of the symptoms, mastitis may develop into two forms: clinical and subclinical. The clinical form is manifested by noticeable changes in the appearance and physicochemical composition of milk, an increase in somatic cell count, and by lesions in the mammary gland, whereas the symptoms of the subclinical form include an increased somatic cell count and the presence of microorganisms in the quarter milk samples [4].

Etiological factors of mastitis may include bacteria, fungi, and viruses. Over 130 bacterial pathogens capable of infecting the mammary gland have been divided into two groups: infectious and environmental bacteria. The first group encompasses microorganisms able to survive inside the mammary gland that with time may also to damage the gland; the group is represented by *Staphylococcus aureus*, *Streptococcus agalactiae*, *Corynebacterium bovis*, and *Mycoplasma spp*. Infections of the pathogens of this group mainly develop as a result of insufficient hygienic standards during milking [5, 6]. The second group of the pathogens is represented by environmental bacteria originating from the natural environment of dairy cattle, e.g., *Escherichia coli*, *Streptococcus uberis* or coagulase-negative staphylococci (CNS). Bacteria classified into this group cannot survive inside the mammary gland.

Prevalence of individual pathogens has been confirmed to be both season- and region-dependent, while investigations monitoring the causes of mastitis in dairy cattle are an important tool that enables the development of targeted preventive programmes [5]. Studies on prevalence of individual aetiological agents in bovine mastitis have already been conducted in the area analysed in the present study [7]; however, these studies did not consider the seasonal character of prevalence and investigated only the subclinical forms of mastitis. Thus, the purpose of the present study was to determine prevalence of individual bacterial pathogens and yeast-like fungi over seven years (2013–2019) in the Warmia Province in cows with the clinical and subclinical forms of mastitis.

## Materials and Methods

Analyses were conducted in 1,665 samples of quarter milk (2013, *n* = 368; 2014, *n* = 350; 2015, *n *= 290; 2016, *n *= 170; 2017, *n *= 173 l 2018, *n* = 224; and 2019, *n* = 90) collected from January 2013 to June 2019 from dairy cattle with clinical or subclinical mastitis originating from various farms located in north-east Poland. The size of the cattle herds varied from 60 to 1,200. All animals were kept in a loose housing barn. During the analysed period, the animals were kept on mats with straw. All samples were collected by the veterinarians responsible for the herd. Samples for the analysis were collected only from the same herds across the years. To the best of our knowledge, the environments have not changed over the analysed period. Subclinical milk samples were included into the study if there were no clinical signs and Somatic cell count (SCC) was higher than 400,000 cells/ml.

Milk samples (0.01 ml) for bacteriological examination were transferred with a calibrated inoculation loop onto the Columbia agar medium supplemented with 5% defibrinated sheep blood (Oxoid, Basingstoke, Great Britain) and onto the Sabouraud medium supplemented with chloramphenicol (Oxoid, Basingstoke, Great Britain). Bacteria were cultured at 37ºC under aerobic conditions for 48 hours, and yeast-like fungi were cultured at room temperature for 7 days. The grown isolates were subjected to microbiological analysis, which included evaluation of the morphology of bacterial colonies, Gram staining, selected biochemical tests (tests for catalase, coagulase and oxidase; API 20E and API 20 NE tests, (bioMérieux, Lyon, France)), CAMP reaction and selected latex tests (Staphytect Plus, PathoDxtra Strep grouping kit (Oxoid)). If more than two types of colonies were detected in a sample, it was considered as contaminated and was not included in subsequent analyses [7–9].

Occurrence of particular aetiological factors of mastitis in individual years of the analysed period was compared. Statistical analysis was carried out using the chi-squared test (GraphPad Prism v. 6.0; GraphPad Software Inc., San Diego, CA, USA). The differences were considered statistically significant at p ≤ 0.05. The seasonal character of occurrence of individual pathogens has not been confirmed; therefore, this part of the analysis was omitted from the results section.

## Results and Discussion

In 2013, mastitis was most commonly induced by *Staphylococcus aureus* (24%), *Streptococcus spp.* (22%), *Streptococcus agalactiae* (12%) and coagulase-negative *Staphylococcus* (CNS) (11%). The other microorganisms were isolated sporadically. No growth of the pathogens was observed in 10% of the samples (Table 1).

**Table 1:** Prevalence of individual pathogens isolated from the milk samples of dairy cattle with clinical mastitis in 2013 (*n* = 368), 2014 (*n* = 350), and 2015 (*n* = 290), 2016 (*n* = 170), 2017 (*n* = 173), 2018 (*n* = 224) and 2019 (*n* = 90)

		*E.* *coli*	*Entero-* *bacteriaceae spp*	*P.* *aeruginosa*	*Streptococcus spp*	*Enterococcus* *Spp*	*S.* *agalactiae*	*S.* *aureus*	CNS	*Corynebacterium spp*	*Trueperella spp*	*Candida spp*	*Pasteurella spp*	no growth
2013	%	4	2	2	22	4	12	24	11	5	2	1	1	10
	n	16	7	7	81	15	44	88	40	18	7	4	4	37
2014	%	5	2	1	25	7	8	18	10	3	1	2	0	18
	n	17	7	4	87	24	28	63	35	11	4	7	0	63
2015	%	10	1	2	43	4	3	6	8	2	0	1	0	20
	n	29	3	6	124	12	9	17	23	6	0	3	0	58
2016	%	5	2	0	44	4	5	11	6	2	1	2	0	18
	n	9	4	0	75	6	8	19	11	4	2	4	0	30
2017	%	8	0,5	0,5	49	3	5	10	4	2	0,5	0,5	0	17
	n	13	1	1	84	6	8	18	7	4	1	1	0	29
2018	%	8	1	1	41	2	2	13	7	6	2	1	0	16
	n	17	2	2	92	5	4	30	16	14	4	3	0	35
2019	%	11	4	1	39	4	4	10	6	1	3	1	0	16
	n	10	4	1	35	4	4	9	5	1	3	1	0	14

In 2014, the most common pathogens isolated from the milk samples were *Streptococcus spp.* (25%), *Staphylococcus aureus* (18%) and CNS (10%) (Table 1).

In 2015–2019, *Streptococcus spp.* were the most often isolated bacteria from the milk samples (above 39%). The other pathogens were isolated occasionally and were detected in less than 15% of all samples analysed during this period (Table 1). In 2014 and 2019, bacteria of the *Pasteurella* genus were not detected, and a successive increase was observed in the percentage of the cases when the analysed microorganisms were not isolated.

**Fig. 1:**
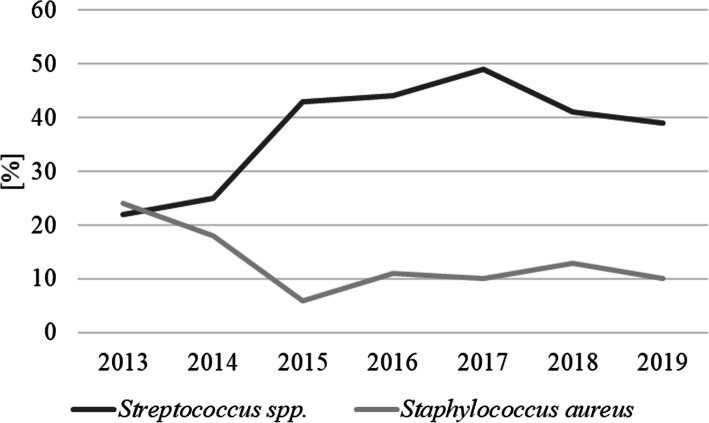
Changes in the trend of etiological factors of mastitis in dairy cattle in north-east Poland between 2013–2019

Over the analysed years, the changes in the dominance of the aetiological factors of mastitis in dairy cattle in north-east Poland were detected. In 2013, *S. aureus* was the most often isolated bacterium; however, in subsequent years, a decrease in incidence of this pathogen was observed. In the case of streptococci, the situation was opposite, and their dominance was observed from 2014 to 2019 (Fig. 1).

Chi-squared test was used to determine significant differences in prevalence of individual pathogens between the years. The differences were considered significant at *P* < 0.05 (Table 2). Unfortunately, in two cases (*Pasteurella* and *Trueperella*), we were unable to perform the test due to inability to meet the test requirements. The differences between the years were statistically significant in the cases of *E. coli* and no growth group; however, in the cases of *Streptococcus* spp, *Streptococcus agalactiae* and *Staphylococcus aureus*, the differences between the years were more pronounced.

**Table 2:** Significant differences in prevalence of individual pathogens isolated from the milk samples of dairy cattle with clinical mastitis in 2013–2019. The differences were estimated by chi-squared test and were considered statistically significant at *p* ≤ 0.05

	*p*-value
*Escherichia coli*	0,028769754 *
*Enterobacteriaceae* spp	0,253668321
*Pseudomonas aeruginosa*	0,439879353
*Streptococcus spp*	3,12916E-14 **
*Enterococcus spp*	0,20726314
*Streptcoccus agalactiae*	2,24517E-06 **
*Staphylococcus aureus*	1,649E-09 **
CNS	0,095020098
*Corynebacterium* spp	0,062552281
*Trueperella* spp	0,161925266
*Candida spp*	0,741015748
*Pasteurella* spp	0,028198293
no growth	0,023202691 *

*p < 0,05, **p < 0,01

Our study aimed to determine prevalence of individual pathogens in the milk samples from the Warmia Province over seven years. These investigations are an important tool that enables the development of optimized preventive programmes. An example of the effectiveness of the implemented protective actions (increasing milking hygiene, udder drying off and blitz therapy) is a significant decrease in the percentage of mammary gland infections induced by *Streptococcus agalactiae*, which is an infectious pathogen considered one of the major aetiological factors of mastitis for many years [5].

In 2013, *Staphylococcus aureus* was the main cause of mastitis in the analysed milk samples. These results are partly consistent with the previously published data obtained in various regions of Poland. The results of the studies carried out in 2002–2013 indicate that bacteria of the *Staphylococcus* genus were the most common cause of mastitis; however, depending on the region or year, CNS or *Staphylococcus aureus* were predominant. Interestingly, a report by Smulski et al. (2006) demonstrated that staphylococci were predominant in large farms, where no apparent problems with the hygienic quality of milk were observed. Therefore, a subsequent reduction in the number of *S. aureus* cases may be due to enhanced milking hygiene, introduction of preventive vaccinations or reduction in the number of flies in the barn [10]. A trend of decreased incidence of mastitis induced by infectious pathogens observed in our study is in agreement with the data of the literature on incidence in other countries [5, 11]. This phenomenon may be due to two reasons. One of the causes may be the fact that the commonly used test for somatic cell count determination in milk performs better in the case of infectious pathogens [12], which considerably facilitates the diagnosis of the disease and accelerates the therapy. The second cause may be enforced implementation of preventive programmes targeted at this group of pathogens at farms located in our study area, which indicates the effectiveness of these programmes.

An increase in the importance of bacteria of the environmental group in the aetiology of mastitis of dairy cows in north-east Poland described in our study is only partially consistent with the findings of other authors. Although environmental pathogens were indicated as the main causative agents of mastitis by most of the authors [5, 11, 13, 14], usually these pathogens are mainly represented by coagulase-negative staphylococci and not by streptococci, which are the main bacteria in our study. This phenomenon may confirm a theory that prevalence of individual bacteria may be local suggesting that unified preventive programmes may sometimes be ineffective. Considering occurrence of streptococci in the area analysed in our study, it may be worthwhile to reconsider the protective strategy targeted at these bacteria. Preventive programmes targeted at the bacteria of the infectious group are effective; however, effectiveness of the programmes targeted at the environmental bacteria is substantially more complex. The main problem is posed by common occurrence of environmental bacteria in the natural environment of dairy cattle (litter and fertilizers), which makes their complete eradication from a farm impossible [15]. According to Makovec and Ruegg (2003), the administration of preparations facilitating the functions of the immune system of the animals (immunomodulators) is advisable to enhance the protection and to facilitate elimination of the pathogen from the organism.

In the case of fungal pathogens, only *Candida* spp. Presence has been confirmed in analysed milk samples. The presence of other yeast-like and mould fungi was not observed. In the area analysed in the present study, fungi appeared sporadically (approximately 1%), which is consistent with the results of other authors [8, 9, 16]. According to Sztachańska et al. (2016), fungal mastitis can result from excessive antibiotic use or poor milking hygiene.

In all study years, growth of pathogens was not detected in some samples of quarter milk received by our laboratory, and the percentage of these samples (10–20%) was comparable with the data obtained in other countries [5, 11, 13, 14]. In these cases, mastitis could have been induced by viruses (bovine herpesvirus types 1 and 4, foot and mouth disease virus and parainfluenza virus type 3) or by bacteria from the genus *Mycoplasma*, which have special growth demands and do not grow on the culture media routinely used in the laboratories. This phenomenon can be explained by low count of an individual pathogen in the mammary gland as a result of immune response or poor pathogen recovery from the samples due to periodical shedding of bacteria [5, 6, 17, 18].

Thus, the role of environmental bacteria has been successively increasing, and the role of infectious pathogens in the aetiology of dairy cattle mastitis has been decreasing in north-east Poland, which may confirm the effectiveness of the implemented preventive programmes and suggests the need for the development of novel scenarios of action targeting environmental pathogens. Regular surveys conducted to monitor regional and seasonal occurrence of aetiological factors of mastitis may contribute to facilitation of the efforts of on-site veterinarians and to minimization of economic losses triggered by this disease. 

## Data Availability

All data generated or analysed during this study are included in this published article.

## Abbreviations

*spp*: Species
CNS: Coagulase negative staphylococcus
CAMP: Christie-Atkins-Munch-Peterson
SCC: Somatic cell count
