# Correction: Streptococci as the new dominant aetiological factors of mastitis in dairy cows in north-eastern Poland: analysis of the results obtained in 2013–2019

**DOI:** 10.1186/s13620-022-00218-5

**Published:** 2022-05-13

**Authors:** E. Kaczorek-Łukowska, J. Małaczewska, R. Wójcik, K. Duk, A. Blank, A. K. Siwicki

**Affiliations:** 1 Department of Microbiology and Clinical Immunology, Faculty of Veterinary Medicine, University of Warmia and Mazury in Olsztyn, Oczapowskiego 13, 10-719 Olsztyn, Poland; 2 Department of Pathophysiology, Forensic Veterinary Medicine and Administration, Faculty of Veterinary Medicine, University of Warmia and Mazury in Olsztyn, Oczapowskiego 13, 10-719 Olsztyn, Poland

**Correction to: Ir Vet J 74, 2 (2022)**

**https://doi.org/10.1186/s13620-020-00181-z**

Following the publication of the original article [1], one of the co-author reported that she changed her name from “K. Naumowicz” to “K. Duk”

The original article has been updated.
